# Magnetic Resonance Imaging Systematically Differs from Histology in Quantifying Macrovesicular Liver Steatosis in Individuals with Morbid Obesity: A Prospective Paired MRI–Histology Study in Bariatric Surgery

**DOI:** 10.3390/diagnostics16091312

**Published:** 2026-04-27

**Authors:** Sergio Carandina, Viola Zulian, Eric Fontas, Antonio Iannelli

**Affiliations:** 1 General and Metabolic Surgery, ELSAN, Clinique Saint Michel, 4, Place du 4 Septembre, 83100 Toulon, France; sergio.carandina@gmail.com (S.C.); zulian.viola@gmail.com (V.Z.); 2 Department of Clinical Research and Innovation, Centre Hospitalier Universitaire de Nice, 4, Avenue Reine Victoria, 06003 Nice, France; fontas.e@chu-nice.fr; 3 General and Metabolic Surgery, Clinique du Parc Impérial, 28 bd de Tzazewitch, 06000 Nice, France

**Keywords:** morbid obesity, metabolic-dysfunction-associated steatotic liver disease, liver histology, magnetic resonance imaging, bariatric surgery, liver steatosis

## Abstract

**Background**: Liver histology remains the gold standard for assessing liver steatosis (LS); however, non-invasive methods are increasingly being explored in clinical practice. This study aimed to evaluate the agreement between magnetic resonance imaging (MRI) and liver histology in quantifying LS in patients with morbid obesity undergoing bariatric surgery (BS). **Methods**: This ancillary study is part of a prospective, double-blind, multicenter, randomized placebo-controlled trial investigating the effects of preoperative omega-3 polyunsaturated fatty acid supplementation on liver volume in morbidly obese patients undergoing BS. The parent trial yielded negative results, and randomization arm was retained as a covariate in all analyses. Patients underwent MRI within 2 days before surgery, followed by intraoperative wedge resection and TruCore needle liver biopsy. Agreement between MRI and histology was assessed using the intraclass correlation coefficient (ICC) and Cohen’s kappa coefficient (K) for both macro- and microvesicular steatosis. **Results**: Thirty-seven patients were enrolled; paired MRI and biopsy data were available for thirty-one (83.8%). Moderate and statistically significant agreement was observed between MRI and both TruCore (ICC: 0.52, *p* = 0.002; K: 0.42, *p* = 0.007) and wedge-resection (ICC: 0.53, *p* = 0.001; K: 0.29, *p* = 0.044) biopsies for macrovesicular steatosis. The MRI-derived values were systematically lower than histological estimates for macrovesicular steatosis (mean MRI: 23.4% vs. histology: 36.7–37.1%). No significant agreement was identified for microvesicular steatosis with either biopsy technique. **Conclusions**: In morbidly obese patients, MRI demonstrates only moderate agreement with liver histology for macrovesicular steatosis and is unreliable for microvesicular steatosis. The systematic underestimation of macrovesicular steatosis by MRI warrants caution when this modality is used as a standalone decision-making tool in this population. Further studies in larger and more heterogeneous cohorts are needed to better define the performance boundaries of MRI-derived fat-fraction measurement across the spectrum of obesity and metabolic liver disease.

## 1. Introduction

Liver steatosis (LS) is defined as the abnormal accumulation of fat in hepatocytes and has become one of the most prevalent liver conditions worldwide. In the absence of secondary causes such as excessive alcohol intake or steatogenic medication, LS falls within the spectrum of metabolic-dysfunction-associated steatotic liver disease (MASLD)—the term now recommended by international consensus to replace non-alcoholic fatty liver disease (NAFLD) [1]. Approximately 20% of individuals with MASLD develop hepatocyte injury and inflammation, a condition referred to as metabolic-dysfunction-associated steatohepatitis (MASH), formerly known as NASH. Of these, a further 20% will progress to advanced fibrosis or cirrhosis [2]. Although LS was historically regarded as a benign condition, recent evidence underscores its strong association with metabolic syndrome, cardiovascular disease, and type 2 diabetes and suggests a potentially causative role in the latter [3,4]. Accurate quantification of LS is, therefore, of direct clinical relevance.

Non-targeted liver biopsy remains the reference standard for histological grading of LS. However, it is an invasive procedure associated with significant sampling variability, patient discomfort, and rare but serious complications including pain, bleeding, and mortality [5]. These limitations have driven interest in non-invasive alternatives. Among imaging modalities, magnetic resonance imaging (MRI)—specifically MRI-derived proton density fat fraction (MRI-PDFF)—has emerged as the most accurate non-invasive tool for LS quantification [6,7]. MRI-PDFF measures the signal-based liver fat fraction, expressed as a percentage, and reflects the proportion of mobile triglycerides within the liver parenchyma [8], a metric that correlates with histological fat quantification, albeit through fundamentally different measurement principles: histology quantifies the proportion of hepatocytes containing visible fat droplets, whereas MRI-PDFF measures the fraction of the total liver proton signal attributable to mobile fat protons—a physical quantity that does not map directly onto cellular morphology. This distinction underlies the systematic discordance between the two methods reported in the present study [6,8].

The histopathological grading system described by Brunt et al. classify macrovesicular steatosis using a 30% threshold to distinguish moderate from mild disease, a criterion that guides clinical decisions such as organ acceptance for liver transplantation or the timing of major liver resections [9,10,11]. MRI-PDFF, however, reports a continuous fat-fraction estimate that does not map directly onto this categorical histological scale [8].

Morbid obesity is closely associated with MASLD, with reported prevalence rates of 80–85% in patients undergoing bariatric surgery (BS) [12,13]. Because serial liver biopsy is not feasible in routine clinical follow-up, MRI represents an attractive tool for longitudinal monitoring of LS evolution after BS. However, the degree of agreement between MRI and histology in this specific population—characterized by extreme adiposity, metabolic comorbidities, and high baseline steatosis—remains incompletely defined. The present study was designed to address this gap by reporting the results of a paired MRI and liver biopsy assessment of LS in a prospective series of morbidly obese patients with metabolic syndrome undergoing BS.

## 2. Materials and Methods

This is an ancillary study to a prospective, double-blind, multicenter, randomized placebo-controlled trial investigating the effects of a 4-week preoperative supplementation with omega-3 PUFA (Omega-3 polyunsaturated fatty acids) on liver left section volume in morbidly obese patients undergoing BS [14], conducted in accordance with the CONSORT guidelines [15]. The parent trial yielded negative results: omega-3 PUFA supplementation did not significantly reduce liver volume compared with placebo. Although omega-3 PUFA may theoretically influence hepatocyte metabolism and MRI signal characteristics through effects on cellular lipid composition, the absence of a significant treatment effect on liver steatosis makes a substantial impact on the present findings unlikely. However, it cannot be entirely excluded that omega-3 PUFA supplementation induced subclinical changes in hepatic lipid composition—specifically a shift in the ratio of unsaturated to saturated fatty acids—that may influence MRI signal characteristics independently of total fat volume, given the known sensitivity of MRI-PDFF to the degree of fatty acid unsaturation [16]. Randomization arm was nonetheless retained as a covariate in all statistical analyses to account for any residual influence.

The study was approved by the Ethics Committee, Comité de Protection des Personnes (CPP) Sud-Méditerranée V (Approval Code: 2016-A00522-49, Approval Date: 8 September 2016), in accordance with French regulations and was conducted in accordance with the Declaration of Helsinki (as revised in 1983). Written informed consent was obtained from all participants. The study is registered at www.clinicaltrials.gov (NCT03006016).

Eligible patients were candidates for BS with a body mass index (BMI) ≥ 35 kg/m^2^ associated with metabolic syndrome as defined by the harmonized international criteria [17], affiliated to the French healthcare system, and willing to provide written informed consent. Exclusion criteria included a prior history of BS, long-term use of corticosteroids or non-steroidal anti-inflammatory drugs, any liver disease other than MASLD, liver cirrhosis, and contraindication to MRI.

### 2.1. Clinical and Biological Work-Up

Physical examination included measurement of blood pressure, body weight (to the nearest 0.5 kg, in light clothing without shoes), height (to the nearest 0.5 cm), and waist circumference (at the midpoint between the lower costal margin and the iliac crest, to the nearest 0.5 cm). After overnight fasting, blood samples were collected for the determination of hemoglobin A1c (HbA1c), fasting glucose and insulin, alanine aminotransferase (ALT), aspartate aminotransferase (AST), gamma-glutamyl transpeptidase (GGT), triglycerides, HDL-cholesterol, LDL-cholesterol, and C-reactive protein (CRP). Insulin resistance was estimated using the homeostatic model assessment of insulin resistance (HOMA-IR), calculated as fasting plasma insulin (mIU/L) multiplied by fasting glucose (mmol/L) divided by 22.5.

### 2.2. Magnetic Resonance Imaging

All MRI examinations were performed using a standardized protocol on a 1.5-T unit (Achieva, Philips, Best, The Netherlands) within 2 days prior to BS. The fat evaluation sequence was a transverse breath-hold low-flip-angle T1-weighted two-dimensional triple-echo spoiled gradient-echo sequence without contrast injection, with the following parameters: repetition time/echo time (ms) of 192/2.46 (in-phase [IP1]), 3.69 (opposed-phase [OP]), and 4.92 (in-phase [IP2]); flip angle 20°; section thickness 6 mm; intersection gap 1.2 mm; matrix 256 × 192; 25 sections; acquisition time 34 s. Fat fraction maps were computed pixel-by-pixel on the MRI workstation (Leonardo VB14, Siemens, Rome, Italy) using the arithmetic mean of the two in-phase images (T2*-corrected), the subtraction of opposed-phase from corrected in-phase images, and division of the latter by the corrected in-phase image multiplied by 50, yielding the liver fat fraction as a direct percentage. Three regions of interest were analyzed, as follows: anterior face of hepatic segment III (corresponding to the site of intraoperative biopsy), segment IV, and segment V [14].

### 2.3. Liver Biopsy

BS was performed within 3 days of MRI. Immediately after trocar placement and prior to the bariatric procedure, two liver biopsies were obtained from hepatic segment III, as follows: needle biopsy using a TruCore device (Argon Medical Devices, Dallas, TX, USA) and wedge biopsy from the edge of the segment. Both specimens were fixed in 4% neutral-buffered formaldehyde, paraffin-embedded, and sectioned at 2 µm for hematoxylin–eosin and Masson’s trichrome staining. Histological assessment was performed by a single dedicated pathologist (SP), blinded to treatment allocation, who quantified the percentage of macrovesicular and microvesicular steatosis for each specimen.

### 2.4. Statistical Analysis

Agreement between MRI and liver biopsy in measuring LS was evaluated using the intraclass correlation coefficient (ICC, two-way mixed effects model, ICC (3,1) in Shrout and Fleiss convention). ICC values < 0.5 indicated poor agreement; values between 0.5 and 0.75, moderate agreement; between 0.75 and 0.90, good agreement; and values ≥ 0.90, excellent agreement [18].

A supplementary categorical analysis was performed by dichotomizing LS at a 30% threshold, distinguishing absent-to-mild steatosis (<30%) from moderate-to-severe steatosis (≥30%), in accordance with established clinical criteria [9]. Agreement was quantified using Cohen’s kappa coefficient (K), interpreted according to Landis and Koch. These thresholds were originally derived in a test–retest reliability framework and their application to inter-method agreement represents an extrapolation; they are nonetheless retained here as the most widely used interpretive benchmarks in comparable method-comparison studies [19]. Two-sided *p*-values < 0.05 were considered statistically significant. All analyses were performed using SAS version 9.1 (SAS Institute Inc., Cary, NC, USA).

## 3. Results

Table 1 summarizes the baseline characteristics of the study population. Of the 37 enrolled patients, paired MRI and liver biopsy data were available for 31 (83.8%), who constituted the analysis population. The mean age was 47.6 ± 12.1 years, 67.6% were female, and mean BMI was 41.4 ± 5.6 kg/m^2^. The most frequent comorbidities were hypertension (56.8%), obstructive sleep apnea (62.2%), dyslipidemia (35.1%), and type 2 diabetes (29.7%).

Mean LS as measured by MRI was 23.43 ± 10.55%. Mean macrovesicular steatosis at wedge biopsy histological analysis was 37.10 ± 26.76%, yielding a statistically significant moderate ICC of 0.53 (95% CI: 0.22–0.74; *p* = 0.0009). Using the 30% cut-off to binarize steatosis, Cohen’s K was 0.29 (95% CI: 0.04–0.55; *p* = 0.0436), corresponding to fair agreement. For TruCore specimens, mean macrovesicular steatosis was 36.71 ± 25.44%, with a moderate ICC of 0.52 (95% CI: 0.18–0.74; *p* = 0.0021) and a K coefficient of 0.42 (95% CI: 0.16–0.67; *p* = 0.0066), indicating moderate categorical agreement. Agreement between the two biopsy methods was close for macrovesicular steatosis (mean difference: 0.39 percentage points), whereas a more substantial discrepancy was observed for microvesicular steatosis (mean difference: 7.56 percentage points, wedge vs. TruCore), consistent with known topographic variability in microvesicular fat distribution. Across both biopsy methods, MRI consistently yielded lower LS estimates than histology (mean difference of approximately 13–14 percentage points for macrovesicular steatosis).

When microvesicular steatosis was considered, mean values were 11.35 ± 8.98% (wedge) and 18.91 ± 16.08% (TruCore) at histology, compared with a mean MRI LS of 22.81 ± 12.55% and 25.72 ± 11.60% respectively. The ICC was 0.29 (95% CI: −0.11–0.60; *p* = 0.0737) for MRI versus wedge biopsy and 0.17 (95% CI: −0.25–0.54; *p* = 0.2126) for MRI versus TruCore—neither reaching statistical significance. K coefficients were equally non-significant (K = 0.17, *p* = 0.1261 for wedge; K = 0.009, *p* = 0.9641 for TruCore). These results collectively indicate the absence of meaningful agreement between MRI and histology for microvesicular steatosis. Detailed concordance statistics are provided in Table 2, and the results of the categorical analysis using the 30% cut-off are summarized in Table 3.

## 4. Discussion

The present study demonstrates that MRI and liver histology yield significantly discordant LS estimates in morbidly obese patients undergoing BS, with only moderate agreement when macrovesicular steatosis is considered and no meaningful agreement for microvesicular steatosis. This finding has direct practical implications: clinicians should interpret MRI-based LS results in this population with appropriate caution, being aware that MRI systematically underestimates macrovesicular steatosis relative to histology by approximately 13–14 percentage points on average. This directional bias was consistent across both independent histological methods, providing convergent evidence of systematic underestimation that does not rely on a single reference technique. For context, an ICC ≥ 0.75–0.80 is generally regarded as the minimum threshold for a measurement tool to be clinically interchangeable with a reference standard, with ICC ≥ 0.90 required for instruments intended to inform individual patient management decisions [18]. The ICC values of 0.52–0.53 observed in the present study fall substantially below this range, providing a quantitative basis for the conclusion that MRI cannot replace histology for LS grading in morbidly obese patients.

The importance of accurately quantifying LS extends across multiple clinical domains. In the context of liver resection, significant steatosis impairs hepatic regenerative capacity and increases postoperative morbidity and mortality, particularly after major resections, through mechanisms including sinusoidal hypoperfusion, impaired hepatocellular energy homeostasis, and activation of pro-inflammatory pathways [20]. In liver transplantation, macrovesicular steatosis exceeding 30% has been established as an independent risk factor for primary non-function, early allograft dysfunction, and post-transplant vascular and biliary complications [21]. Beyond these surgical applications, MASLD is increasingly recognized as a multisystem disease associated with metabolic syndrome, cardiovascular events, type 2 diabetes, chronic kidney disease, and reduced pulmonary function [3,22,23]. The ability to reliably quantify LS non-invasively, therefore, carries important implications for risk stratification and treatment decision making.

Among non-invasive imaging modalities, MRI-PDFF has emerged as the most validated technique for LS quantification [7]. A meta-analysis by Gu et al. reported area under the ROC curve values ≥ 0.90 for MRI-based LS grading, with particularly high accuracy (AUROC = 0.98) in distinguishing grade 0 from grade 1–3 steatosis [24]. A prospective study of 28 patients undergoing hepatic resection reported 100% accuracy for MRI in identifying > 30% steatosis preoperatively [25], and an ex vivo evaluation of cadaveric donor livers demonstrated moderate correlation between MRI-PDFF and macrovesicular steatosis [26]. Our results in morbidly obese patients are consistent with this moderate level of agreement; however, the systematic underestimation of macrovesicular steatosis by MRI warrants specific attention in this population. The extreme adiposity, altered hepatic fat distribution, and metabolic derangements characteristic of morbid obesity may contribute to this discordance, and extrapolation of MRI performance data from general or lean populations is not straightforward.

The absence of agreement between MRI and histology for microvesicular steatosis is a clinically meaningful finding. Although the prognostic implications of microvesicular steatosis remain less clearly defined than those of macrovesicular disease, available evidence on its prognostic significance remains inconclusive. Elevated microvesicular steatosis in grafts with concurrent macrovesicular disease has been associated with worse liver transplantation outcomes in some series [27], whereas others found no significant impact [28]. A pathological study of MASLD patients further documented an association between microvesicular steatosis and hepatocellular ballooning injury and megamitochondria [29]. Given this residual uncertainty and the demonstrated inability of MRI to quantify microvesicular steatosis in the present cohort, histological assessment remains indispensable when microvesicular disease is a clinical consideration. The absence of MRI–histology agreement for microvesicular steatosis warrants mechanistic consideration. MRI-PDFF quantifies the proton density fat fraction by measuring the signal contribution of mobile triglycerides within hepatic tissue, and microvesicular fat—which consists of small cytoplasmic lipid droplets—does contain mobile triglycerides that are in principle detectable by this technique. The lack of agreement observed here is, therefore, not attributable to signal invisibility, but rather to several converging factors. Microvesicular fat droplets are individually small and widely dispersed within the cytoplasm, yielding a lower volumetric fat concentration per hepatocyte than macrovesicular disease; this may result in a weaker, more diffuse signal that is difficult to resolve reliably at the field strength employed. Furthermore, MRI-PDFF integrates total fat signal across each voxel without distinguishing macrovesicular from microvesicular components—it reports an aggregate fat fraction that cannot be decomposed into histological subtypes. The histological assessment, by contrast, quantifies each pattern separately, creating a fundamental measurement mismatch. Finally, histological grading of microvesicular steatosis is itself subject to considerable inter- and intra-observer variability, dependent on staining protocol and magnification, which introduces noise into the reference standard independent of MRI performance. These combined factors explain why MRI-PDFF, despite its sensitivity to mobile triglycerides in aggregate, cannot reliably serve as a surrogate for the histological microvesicular steatosis grade.

Several methodological strengths merit comment. Data were prospectively collected within the framework of a rigorous randomized controlled trial, ensuring high-quality paired measurements. The use of two concurrent biopsy techniques—TruCore needle and wedge resection—provides a more comprehensive histological reference than either method alone and allowed internal consistency checks. The MRI protocol was standardized across all participants and performed on a single scanner, limiting technical variability. Regarding the potential influence of the omega-3 PUFA intervention on MRI-derived liver fat measurements, the parent trial demonstrated no significant effect of supplementation on liver volume or steatosis, and randomization arm was retained as a covariate in analyses. Any residual effect on hepatocyte lipid composition or MRI signal characteristics is therefore unlikely to have materially biased the present results.

The principal limitation of this study is that it is an ancillary analysis of a trial powered for a different endpoint—liver volume reduction—and no independent sample size calculation was performed for the assessment of MRI–histology agreement. The resulting sample of 31 patients with complete data must, therefore, be considered underpowered for the present purpose. The wide confidence intervals observed throughout—notably ICC 0.22–0.74 for macrovesicular steatosis versus wedge biopsy and Kappa 0.04–0.55 for the corresponding categorical analysis—reflect this limitation directly and should caution against overinterpretation of the point estimates. The study population was also highly selected—patients with morbid obesity and documented MASLD fulfilling metabolic syndrome criteria—which restricts generalizability to lean or overweight individuals and to patients with MASLD in the absence of metabolic syndrome. The MRI protocol employed—a three-echo spoiled gradient-echo sequence with arithmetic fat-fraction computation on a 1.5-T unit—differs from the multi-echo Dixon and MR spectroscopy-based MRI-PDFF protocols now considered the reference standard in the literature. Modern multi-echo approaches provide more comprehensive correction for T2* decay and fat spectral complexity, and may yield superior agreement with histology; direct comparison of our results with studies employing these newer protocols should, therefore, be made with caution. Furthermore, histological assessment was performed by a single pathologist, and no inter- or intra-observer variability data are available; this represents a limitation of the reference standard that may have introduced unquantified measurement error into the agreement analyses. Finally, the cross-sectional design does not permit conclusions regarding the trajectory of MRI-histology agreement after bariatric-metabolic surgery.

A noteworthy internal discrepancy was observed between the two histological techniques for microvesicular steatosis: mean values were 11.35% for wedge biopsy and 18.91% for TruCore needle biopsy. This difference is consistent with the well-established phenomenon of subcapsular sparing, whereby the superficial hepatic parenchyma sampled by wedge resection tends to exhibit lower degrees of steatosis—particularly microvesicular steatosis—compared with the deeper tissue sampled by needle biopsy [14]. For macrovesicular steatosis, by contrast, both techniques yielded closely concordant estimates (37.10% vs. 36.71%), supporting the internal consistency of the histological reference for the primary endpoint of this study. Importantly, despite their quantitative divergence for microvesicular steatosis, both biopsy methods independently demonstrated the absence of meaningful agreement with MRI, converging on the same conclusion from anatomically distinct sampling sites. This convergence strengthens the inference that MRI cannot reliably quantify microvesicular steatosis in morbidly obese patients, irrespective of the histological reference employed. Nonetheless, as neither biopsy technique fully represents whole-liver fat content, the inherent limitations of any sampling-based reference standard must be acknowledged when interpreting agreement statistics [30].

The present findings should not be interpreted as a general indictment of MRI-PDFF, which has demonstrated excellent diagnostic performance across a wide range of populations and field strengths in the existing literature. Our conclusions are strictly limited to morbidly obese patients with MASLD undergoing bariatric surgery, in whom the unique metabolic and morphological context may compromise MRI performance in ways that are not captured by studies conducted in leaner or more heterogeneous cohorts.

## 5. Conclusions

In morbidly obese patients with MASLD undergoing bariatric surgery, MRI demonstrates only moderate agreement with liver histology for macrovesicular steatosis and systematically underestimates its severity relative to histological examination. No meaningful agreement was identified for microvesicular steatosis.

Based on the present findings, MRI-derived fat-fraction measurement cannot be recommended as a standalone decision-making tool for liver steatosis grading in morbidly obese patients with MASLD undergoing bariatric surgery; these conclusions should not be extrapolated to other populations in which MRI-PDFF has demonstrated high diagnostic performance. These results underscore the continued need for histological assessment in settings where precise LS quantification influences clinical management, and highlight the importance of future studies in larger, more heterogeneous cohorts to better characterize the performance boundaries of MRI-PDFF across the full spectrum of obesity and metabolic disease.

## Figures and Tables

**Table 1:** Baseline patient characteristics. Values are means ± SD unless otherwise indicated.

Parameter	Study Population (*n* = 31)
Age (years)	47.6 ± 12.1
Sex female, *n* (%)	67.6
Weight (kg)	113.9 ± 18.9
Height (cm)	165.9 ± 9.8
BMI (kg/m^2^)	41.4 ± 5.6
Systolic blood pressure (mmHg)	133.8 ± 17.4
Diastolic blood pressure (mmHg)	78.5 ± 15.2
Waist circumference (cm)	126.5 ± 15.2
Sleep apnea syndrome, *n* (%)	62.2
Type 2 diabetes, *n* (%)	29.7
Dyslipidemia, *n* (%)	35.1
Hypertension, *n* (%)	56.8
AST (U/L)	35.1 ± 17.4
ALT (U/L)	48.8 ± 26.0
GGT (U/L)	52.4 ± 42.9
Total cholesterol (mmol/L)	5.2 ± 1.3
HDL cholesterol (mmol/L)	1.2 ± 0.3
LDL cholesterol (mmol/L)	3.1 ± 1.1
Triglycerides (mmol/L)	2.1 ± 1.5
Fasting glycemia (mmol/L)	6.7 ± 2.1
C-peptide (nmol/L)	1.4 ± 1.2
Glycated hemoglobin (%)	6.3 ± 0.9
Insulin (mU/L)	36.4 ± 40.8
HOMA-IR	12.0 ± 18.5
C-reactive protein (mg/L)	7.9 ± 6.8

BMI, body mass index; AST, aspartate aminotransferase; ALT, alanine aminotransferase; GGT, gamma-glutamyl transpeptidase; HDL, high-density lipoprotein; LDL, low-density lipoprotein; HOMA-IR, homeostasis model assessment of insulin resistance.

**Table 2:** Agreement between MRI-derived and histology-derived liver steatosis estimates.

Comparison	MRI LS Mean ± SD (%)	Histology LS Mean ± SD (%)	Mean Difference (MRI − Hist.)	ICC (95% CI)	*p*-Value
Macrovesicular steatosis					
MRI vs. wedge biopsy	23.43 ± 10.55	37.10 ± 26.76	−13.67%	0.53 (0.22–0.74)	0.0009
MRI vs. TruCore biopsy	23.43 ± 10.55	36.71 ± 25.44	−13.28%	0.52 (0.18–0.74)	0.0021
Microvesicular steatosis					
MRI vs. wedge biopsy	22.81 ± 12.55	11.35 ± 8.98	+12.08%	0.29 (−0.11–0.60)	0.0737
MRI vs. TruCore biopsy	25.72 ± 11.60	18.91 ± 16.08	+4.52%	0.17 (−0.25–0.54)	0.2126

LS, liver steatosis; ICC, intraclass correlation coefficient; CI, confidence interval; MRI, magnetic resonance imaging. Mean difference = MRI-derived value minus histology-derived value; negative values indicate underestimation by MRI, positive values indicate overestimation.

**Table 3:** Categorical agreement between MRI and liver histology using a 30% liver steatosis threshold.

	MRI ≥ 30%	MRI < 30%	K	*p*-Value
Macrovesicular steatosis				
Wedge biopsy ≥ 30%	8 (25.8%)	11 (35.5%)	0.29	0.044
Wedge biopsy < 30%	1 (3.2%)	11 (35.5%)		
TruCore biopsy ≥ 30%	11 (35.5%)	10 (32.3%)	0.42	0.007
TruCore biopsy < 30%	0 (0%)	10 (32.3%)		
Microvesicular steatosis				
Wedge biopsy ≥ 30%	1 (3.8%)	0 (0%)	0.17	0.126
Wedge biopsy < 30%	7 (26.9%)	18 (69.2%)		
TruCore biopsy ≥ 30%	2 (8.7%)	3 (13.0%)	0.009	0.964
TruCore biopsy < 30%	7 (30.4%)	11 (47.8%)		

Values are *n* (% of total analysis population). K, Cohen’s K.

## Data Availability

The data supporting the findings of this study are available from the corresponding author upon reasonable request.
